# Epithelial-to-mesenchymal transition in cancer: complexity and opportunities

**DOI:** 10.1007/s11684-018-0656-6

**Published:** 2018-07-24

**Authors:** Yun Zhang, Robert A. Weinberg

**Affiliations:** 1 Whitehead Institute for Biomedical Research;; 2 MIT Department of Biology;; 3 Ludwig/MIT Center for Molecular Oncology, Cambridge, MA 02142, USA

**Keywords:** epithelial-to-mesenchymal transition, cancer, metastasis, cancer stem cell

## Abstract

The cell-biological program termed the epithelial-to-mesenchymal transition (EMT) plays an important role in both development and cancer progression. Depending on the contextual signals and intracellular gene circuits of a particular cell, this program can drive fully epithelial cells to enter into a series of phenotypic states arrayed along the epithelial-mesenchymal phenotypic axis. These cell states display distinctive cellular characteristics, including stemness, invasiveness, drug-resistance and the ability to form metastases at distant organs, and thereby contribute to cancer metastasis and relapse. Currently we still lack a coherent overview of the molecular and biochemical mechanisms inducing cells to enter various states along the epithelial-mesenchymal phenotypic spectrum. An improved understanding of the dynamic and plastic nature of the EMT program has the potential to yield novel therapies targeting this cellular program that may aid in the management of high-grade malignancies.

## EMT: a naturally occurring transdifferentiation program

### Basics of the EMT program

**Fig. 1:**
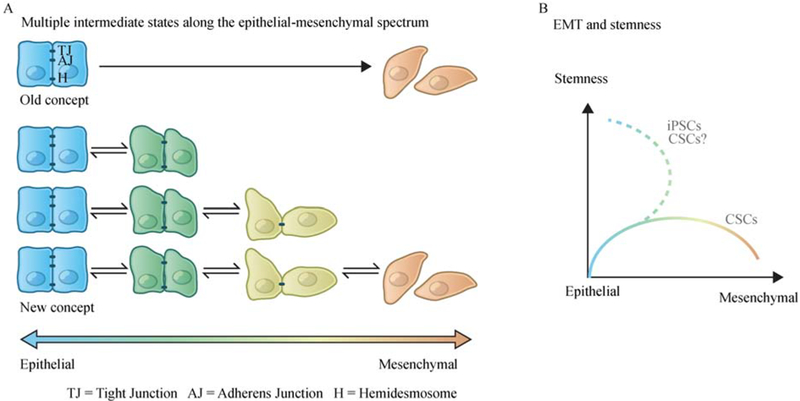
The dynamic and plastic nature of the EMT program. (A) Rather than a unidirectional binary switch between two distinct cell states, accumulating evidence suggests that the epithelial-to-mesenchymal transition (EMT) program generates a spectrum of different intermediate cell states between the extreme epithelial and mesenchymal endpoints. (B) Activation of EMT program is associated with the entrance into stem cell programs, though in certain contexts, constitutive activation of an EMT program in carcinoma cells leads to the loss of stem-like properties. Cancer cells undergone a sequential EMT-MET reprogramming could be very different from the original epithelial cells in the primary tumor. When reprogramming somatic cells into induced pluripotent stem cells (iPSCs), sequential introduction of Yamanaka factors in a specific order (first OCT-4 with KLF4, then c-MYC, and finally SOX2), rather than the simultaneous exposure, has been found to significantly improve the reprogramming efficiency. In this specific protocol, a sequential EMT-MET state change has been observed, showing an intermediate state with upregulated EMT-TFs and enhanced mesenchymal characteristics before entering the epithelial pluripotent state [112]. It is plausible that a similar sequential EMT-MET transition could generate cancer cells with increased stemness and the ability to form macro-metastatic colonies.

The epithelial-to-mesenchymal transition (EMT) is a cell-biological program that naturally occurs in a broad range of tissue types and developmental stages. As its name implies, the EMT program converts epithelial cells to cells that have entered into more mesenchymal cell states arrayed along the epithelial (E) versus mesenchymal (M) axis. Depending on the contextual signals received by a cell within a tissue and the intracellular gene circuitry of the cell, this program generates cells that enter into a series of intermediate phenotypic states arrayed along the E-M axis and, when driven to its extreme, converts a fully epithelial cell to one residing in a fully mesenchymal cell state (Fig. 1A) [1]. Profound biological differences distinguish the extreme poles of the epithelial versus mesenchymal axis: the epithelial cells exhibit epithelial cell-to-cell junctions and the apical-basal polarity, while the mesenchymal cells exhibit a heightened motility and invasiveness with spindle-like morphology that lacks apical-basal polarity [1,2].

Initially reported by Elizabeth Hay in 1982 [3], the EMT program is now known to have essential roles in multiple steps of embryonic morphogenesis [1,4]. This program operates during development to ensure the interconversions of cells that are required to form distinct cell types in metazoans. As examples, an EMT program gives rise to the mesoderm and primary mesenchyme from the primitive streak during gastrulation as well as to migratory neural crest cells. Disrupting this program in transgenic mice by silencing expression of certain transcription factors that orchestrate EMT programs (EMT-TFs), results in severe developmental defects [5–9]. Of note, while we refer here to “the EMT program”, we also acknowledge that there are multiple versions of this program, depending on the EMT-TFs that are orchestrating this state change within a cell, the cell type in which it is occurring, and the microenvironment in which this cell resides.

In addition, the EMT program plays an essential role in various pathological processes, including wound healing, tissue fibrosis, and cancer progression [1,10]. In all of these processes, EMT and the reverse of this program, termed mesenchymal-to-epithelial transition (MET), induces multiple fundamental changes in cell physiology in addition to the morphologic differences noted above. For example, during epithelial wound healing, the viable epithelial cells at the edge of the wound site undergo a partial EMT in order to gain motility and move as a coordinated group of cells to help reconstruct the epithelial cell sheet. In the end, the quasi-mesenchymal cells created by the activation of an EMT program then revert to their epithelial phenotype through an MET to reestablish the epithelial sheet integrity [11]. Of note, in all of these pathological conditions, epithelial cells can activate the multifaceted EMT program to various extents, often acquiring many, but not all, of the traits associated with fully mesenchymal cells. Stated differently, under physiological conditions, epithelial cells activate an EMT program rarely if ever progress into an entirely mesenchymal cell state. Of note, EMT programs operate in both normal epithelial cells and their neoplastic derivatives.

The importance of EMT program in tumor progression has been established in the past two decades with a rapidly growing number of studies demonstrating the activation of EMT programs during the process of malignant progression [1,10,12]. In carcinomas, in which the contributions of the EMT program to cancer cell phenotypes have been most intensively studied, EMT-induced mesenchymal traits enable carcinoma cells to complete many of the steps of the invasion-metastasis cascade, including the local invasion of neoplastic cells at the primary tumor site, their intravasation into blood vessels, translocation through the circulation, extravasation into the parenchyma of distant tissues, and survival as micrometastatic deposits [13,14]. As noted above in the context of normal cells, it is also rare for carcinoma cells to lose all epithelial traits and gain a full spectrum of mesenchymal characteristics. An important though rare exception is provided by carcinosarcomas, in which distinct epithelial and mesenchymal compartments coexist and are derived from a common cellular precursor [15].

In addition, as described above in the context of normal epithelial cells, the EMT program is often activated reversibly, permitting the carcinoma cells to revert back to more epithelial states via MET, doing so in certain cellular contexts [16]. This plasticity of cell phenotypes may play an essential role in the last step of the metastatic cascade, the outgrowth of disseminated micrometastatic deposits into macroscopic metastases, the process termed colonization; as shown in mouse models and patient-derived xenograft (PDX) models, activation of an EMT program is crucial for the dissemination of tumor cells, whereas the disseminated cells need to undergo MET in order to efficiently form macroscopic metastases [17–19]. Nonetheless, we note that successful colonization process may not be within the powers of the EMT program, but instead may depend on tissue-adaptive programs that disseminated cancer cells contrive after arriving in distant, unfamiliar tissue microenvironments. This dynamic and plastic nature of the EMT program during the progression of carcinomas should influence the interpretation of EMT-associated observations from both pre-clinical and clinical studies.

### EMT and stemness

The concept of cancer stem cells (CSCs) in carcinomas is based on the observations that phenotypically distinct subpopulations of cancer cells coexist within a single tumor, with a small number of cancer cells showing certain similarities with non-neoplastic stem cells, including enhanced self-renewal and an ability to regenerate the entire neoplastic tumor tissue including more differentiated non-CSC derivatives [10,20,21]. In this paradigm, CSCs are hypothesized to play essential roles in continued tumor growth, metastasis initiation, drug resistance, and tumor relapse after therapy. An interesting but previously unanticipated aspect of the EMT program is its association with the entrance into epithelial stem cell programs (Fig. 1B) [22–24]. Since the initial discovery of the connection between breast cancer cells that have undergone an EMT and their entrance into a stem-cell like state [22,24], a number of studies have reported that acquisition of stemness following the activation of an EMT program in multiple cancer types including pancreatic, prostate, colorectal, ovarian cancer among other types of carcinomas [25–28].

A fact, we now know, at least in the context of mammary epithelial tissue, is that the association between EMT and stemness holds true for both normal and neoplastic conditions [22,23]. EMT-TF SLUG (also known as SNAI2) is expressed in the basal and/or abluminal layer of normal murine and human mammary ducts at sites where normal mammary stem cells are proposed to reside. (The functional assay for normal mammary stem cells involves implanting candidate mammary epithelial cells (MECs) into mammary fat pads from which the resident endogenous MECs have been surgically removed; stemness is judged by the ability of the implanted cells to generate entire mammary ductal trees after a number of weeks.) Forcing a population of normal MECs through an EMT, achieved via transiently expressing SLUG and SOX9, dramatically increased the representation of mammary stem cells within the heterogeneous populations of MECs. Conversely, shutdown of SLUG in MECs deprives them of their mammary gland-reconstituting activity, indicating a causal role of this EMT-TF in the entrance into and/or maintenance of the stem cell state [23]. In parallel experiments, forcing bulk populations of human breast cancer cells through an EMT program by transient expression of EMT-TFs can increase the frequency of tumor-initiating CSCs, as shown by the increased expression of CSC-specific cell-surface markers, an elevated ability to form tumorspheres in suspension culture, and an elevated ability to initiate tumors in immuno-compromised mice [22]. It is still unclear precisely how different the EMT-induced stem cell programs are between the non-neoplastic cells and their corresponding neoplastic cells. However, we now know that in mammary gland, normal gland-reconstituting stem cells and breast CSCs depend on the actions of the related EMT-TFs Slug and Snail, respectively, suggesting that the stem-cell programs operating in CSCs and normal stem cells of the corresponding normal tissue are likely to differ significantly in their details [29].

The association between the EMT program and the CSC state, as described above, suggests that, in general, activation of this program in non-CSCs enables their conversion into CSCs. Indeed, populations of non-CSC have been shown to spontaneously undergo EMT under appropriate conditions, acquiring CSC-like cell-surface markers and an enhanced capacity to seed tumors in mice [30,31]. The resulting CSCs, because of their functioning as stem cells, have the ability to generate more differentiated non-CSC derivatives, possibly through the activation of an MET. Of note, the substantial phenotypic plasticity between non-CSCs and CSCs, determined by the dynamic and plastic nature of the EMT program during cancer progression, suggests that in neoplastic tissues, a unidirectional stem-cell hierarchy does not apply. Instead, a description of bidirectional interconversions involving phenotypic shifts between distinct cellular states appears to be more appropriate.

It remains unclear precisely how EMT programs facilitate the entrance of both normal and neoplastic epithelial cells into stem cell states. Independent of this issue is a second one: where do stem cells reside along the spectrum of phenotypic states that ranges from fully epithelial to fully mesenchymal? Several recent studies reported that in certain contexts, constitutive activation of an EMT program in carcinoma cells leads to the loss of stem-like properties, suggesting that the acquisition of mesenchymal traits is not always associated with the acquisition of increased stemness [17,18,32]. Several possible explanations of this observation are worthy of further exploration: (1) expression of certain transcription factors or other types of gene regulators, similar to Sox9 in differentiated mammary luminal/myoepithelial cells, might be required in order to coordinate an EMT program that leads cells into stem cell states; (2) stemness and mesenchymal traits may represent two mutually exclusive sets of cellular characteristics; (3) stemness is only associated with residence in certain intermediate transition states along the E-M axis, dictating that only those cells that express certain combinations of epithelial and mesenchymal traits acquire stem-like properties [33].

## EMT in cancer progression

### EMT and metastasis

**Fig. 2:**
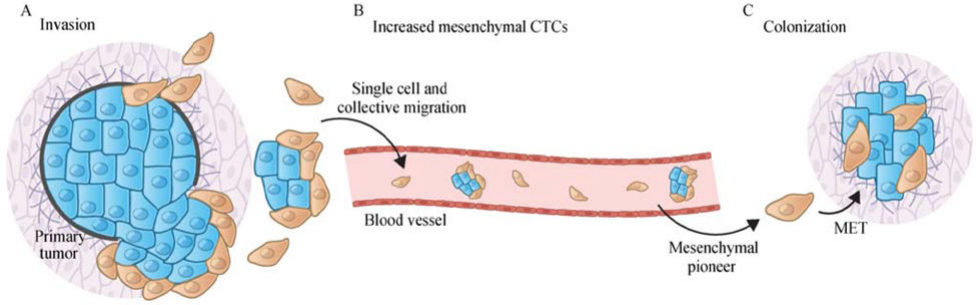
The EMT program facilitates multiple steps of the invasion-metastatic cascade. (A) At the primary tumor site, induction of an EMT program allows carcinoma cells to lose cell-cell junctions, degrade local basement membrane via elevated expression of various matrix-degrading enzymes and supports cancer cell dissemination in both the “single cell” and “collective migration” modes. (B) Many circulating tumor cells (CTCs), representing carcinoma cells that have entered into the vasculature and may thereafter be capable of seeding new metastatic colonies at distant anatomical sites, display partial EMT activation with co-expression both epithelial and mesenchymal markers. Moreover, mesenchymal CTCs have been found to be significantly enriched in cancer patients with refractory or progressive disease [45]. (C) At the colonization step, robust outgrowth of macro-metastases, at least in some destination organ sites, requires the reversion of EMT program and the associated gain of epithelial characteristics.

Metastasis remains the most life-threatening risk for cancer patients, with more than 90% of cancer-associated deaths being caused by metastatic disease rather than by the corresponding primary tumors [13,34]. The increased motility/invasiveness associated with the mesenchymal cell state has linked the EMT program with metastasis, in which cell separation from the primary tumor mass can be considered as the first step of the invasion-metastasis cascade. Thus, a variety of studies using both mouse models and cultured human cancer cells have demonstrated that induction of an EMT program allows carcinoma cells to lose cell-cell junctions, degrade local basement membrane via elevated expression of various matrix-degrading enzymes, and thus support their migration and invasion as single cells (Fig. 2A) [17,18,35,36]. In an alternative mode of cancer cell invasion, cohorts of cells, rather than single cells, migrate together into adjacent tissues. In fact, this “collective migration” mode might be even more frequent than single-cell dissemination in clinical tumors, as supported by several recent studies showing the polyclonal nature of many invasive/metastatic colonies [37–40]. Although cells residing within the bulk of these invasive masses usually maintain cell-cell junction and express E-cadherin, the prototypical marker of epithelial cell state, detailed analyses of these invasive cohorts suggest that carcinoma cells at the leading edges of these invasive masses often express certain mesenchymal characteristics, supporting the notion in which invading leader cells undergo EMT to gain motility and release various proteases to degrade the extracellular matrix, doing so in order to pave the way for all the more epithelial follower cells (Fig. 2A) [29,41–43]. In addition, E-cadherin has been reported to exist in functionally distinct complexes within the same cell [44], though it remains unclear how these different complexes contribute to cancer progression and whether an EMT program regulates the switch of E-cadherin between these complexes. At present, key experimental tests to demonstrate the essentiality of EMT for invasiveness, which would depend on completely blocking this program and demonstrating continued invasion, have not yet been produced.

In addition to local invasiveness, the association between EMT and entrance into the CSC state suggests that this program could also contribute to additional steps in the invasion-metastasis cascade. Thus, the possession of tumor-initiating powers of CSCs would seem to be a critical prerequisite to the founding by disseminated cancer cells of metastatic colonies. Many circulating tumor cells (CTCs), representing carcinoma cells that have invaded into the vasculature (intravasated) and may thereafter be capable of seeding new metastatic colonies at distant anatomical sites, display partial EMT activation with coexpression of both epithelial and mesenchymal markers (Fig. 2B) [45]. Of note, in many of the published analyses of CTCs, the enrichment methods that were employed were based on the display of certain epithelial cell-surface markers such as EpCAM, and thus may fail to capture cancer cells that have proceeded more extensively through the EMT program, causing them to lose the bulk of their epithelial cell-surface markers. In any event, only rare cells among the CTCs may eventually serve as the founders of metastatic colonies because of the profound inefficiency of the post-dissemination colonization process [13].

Disseminated cancer cells, which have traveled to distant sites and invaded the parenchyma of various tissues, may initially enter into a dormant state due to their inability to adapt to a newly encountered tissue micro-environment. The behavior of disseminated cancer cells is of great interest because such cells can potentially proceed to form macroscopic metastatic colonies, the most deadly phase of malignant cancer progression, but this represents a high barrier to successful colonization and might provide a therapeutic time window to manage metastatic disease. Following extravasation, disseminated tumor cells almost always become eliminated or, alternatively, enter a state of dormancy in the ostensibly inhospitable newly encountered tissue microenvironment [46–48]. In clinical practice, patients successfully treated for their primary tumors may often harbor disseminated cancer cells residing in a dormant state in various organs throughout the body. This apparent dormancy may result from their inability to proliferate in their new microenvironments or, alternatively, they may exhibit a low proliferation rate in which any increases in the number of carcinoma cells is counter-balanced by continued immune attacks [49]. Although it is difficult to rigorously prove that metastatic colonies are directly developed from these dormant disseminated cells, the presence of dormant tumor cells in the bone marrow has been found to correlate significantly with clinical recurrence in breast cancer patients [50]. In addition, in both mouse models and patient samples, the expression of EMT-associated traits and the systemic dissemination of cancer cells have been found to begin early in the disease course, being evident even in certain pre-neoplastic lesions [29,51–53]. These findings indicate that it is of great clinical importance to eliminate these dormant cancer cells or to prevent their development into macro-metastases.

Interestingly, it seems clear that the robust outgrowth of metastatic colonies, at least in some destination organ sites, requires the plasticity between E versus M states rather than constitutive residence in a fully mesenchymal state (Fig. 2C). For example, studies using mouse models of breast and skin cancers have demonstrated that activation of an EMT program is important for primary tumor cells to disseminate into the lungs, while the disseminated cells need to subsequently reverse the EMT program and gain epithelial characteristics in order to efficiently form macroscopic metastases [17,18]. Similar observations have been made in mouse models of pancreatic cancer where cancer cells form metastases in the liver [54]. It remains unclear why the formation of macrometastases seemingly founded by more mesenchymal pioneers requires the subsequent generation by these pioneers of more epithelial progeny. Nonetheless, we note that restoration of the full epithelial traits is not necessary for all types of metastases, as shown in most cases of invasive lobular carcinoma of the breast, in which E-cadherin expression is completely lost due to CDH1 mutation but macrometastases can still be formed [55].

### EMT contributes to tumor heterogeneity

The phenotypic diversity of neoplastic cells within a tumor is increasingly considered as a major obstacle to the success of cancer therapies [56–59]. Both genetic and epigenetic mechanisms contribute to phenotypic heterogeneity within individual tumors. With recent advances in next-generation sequencing technologies, the impact and influence of genetic heterogeneity in designing effective treatment strategies have been widely recognized and extensively discussed [60]. Still, the ability of cancer cells with the same genetic profile to interconvert between multiple distinct phenotypic states via epigenetic regulatory mechanisms, thereby contributing to critical cancer cell behaviors, has not been fully understood.

The ability of the EMT program in generating invasive mesenchymal cells and CSCs represents an important example how epigenetic mechanisms contribute to forming tumor heterogeneity. Similarly, expression of oncogenic mutant PIK3CA^H1047R^ in mouse mammary epithelial cells has been shown to evoke cell dedifferentiation into a multipotent stem-like state and thereby facilitate the formation of a heterogeneous, multi-lineage mammary tumor [61]. However, it remains unclear whether this program has any connections with the EMT-induced stem cell programs. In another example, loss of RB1 and TP53 function in prostate adenocarcinomas enables lineage plasticity of androgen receptor (AR)-dependent luminal epithelial cells, allowing them to shift into AR-independent basal-like cells, and thus gain resistance to targeted anti-androgen drugs [62,63]. In both cases, certain mutant oncogenes or loss of tumor suppressors induce cancer cell plasticity, generating different daughter cells at the epigenetic level with distinct cellular behaviors in drug resistance, tumor initiation and metastasis. A number of intermediate EMT states have also been identified in transgenic mouse models of skin squamous cell carcinoma and breast cancer. These EMT subpopulations coexist in the primary tumor but display differences in their invasiveness and metastatic potentials [64]. All these examples reveal the critical need to further explore these plasticity mechanisms in the context of cancer in order to develop effective anti-cancer therapies in the future.

### EMT confers therapeutic resistance

**Fig. 3:**
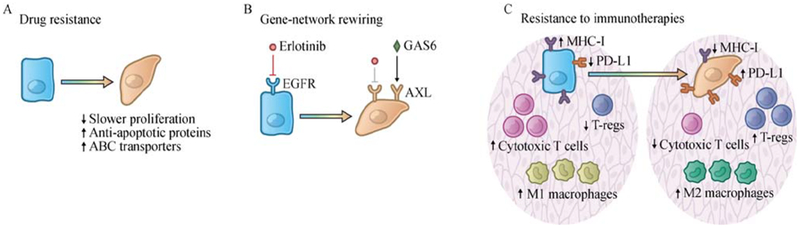
EMT confers therapeutic resistance. (A) EMT confers multidrug resistance on cancer cells. EMT-induced multidrug resistance involves a number of mechanisms, including a slow proliferation rate, elevated expression of anti-apoptotic proteins, and upregulation of ATP binding cassette (ABC) transporters that mediate drug efflux. (B) The E-to-M transition may induce cancer cells into novel phenotypic states and make certain therapeutic targets dispensable for continued cell viability. For example, the E-to-M transition switches the dependence of carcinoma cells from the EGFR to the AXL receptor tyrosine kinase in non-small-cell lung cancer cells, thereby yielding resistance to EGFR-targeted therapy. (C) The EMT program contributes to the establishment of an immunosuppressive tumor microenvironment and confers resistance to immunotherapies. In a cell-autonomous manner, induction of EMT program in carcinoma cells downregulates MHC-I molecules and β2-microglobulin while upregulating PD-L1. In addition, induction of EMT program leads to various non-cell-autonomous changes, remodeling the tumor microenvironment by recruiting M2 (pro-tumorigenic) macrophages and T-regs, and suppressing the infiltration of cytotoxic T cells.

EMT programs increase the resistance to cell death induced through various mechanisms both in embryos and in cancer cells. For example, accumulating evidence indicates that activation of EMT confers multidrug resistance on cancer cells [10,65]. Similar to certain non neoplastic tissue stem cells that exhibit higher levels of resistance to multiple chemotherapeutic agents, EMT-induced multidrug resistance involves a number of mechanisms, including a slow proliferation rate, elevated expression of anti-apoptotic proteins, and upregulation of ATP binding cassette (ABC) transporters that mediate drug efflux (Fig. 3A) [66–68]. In addition, the E-to-M transition may be associated with entrance into novel phenotypic states, which explains, in turn, the acquired resistance to many targeted therapies once the therapeutic targets become dispensable for continued cell viability. For example, in non-small-cell lung cancer (NSCLC) and ovarian cancer, the E-to-M transition switches the dependence of carcinoma cells from the EGFR to the AXL receptor tyrosine kinase, thereby yielding resistance to EGFR-targeted therapy (Fig. 3B) [69,70]. Moreover, the survival of relatively slow-cycling subpopulations of carcinoma cells may allow certain partially mesenchymal cancer cells to accumulate additional genetic mutations, ultimately generating highly proliferative descendants exhibiting acquired phenotypic advantages such as drug resistance. Indeed, several recent studies have shown the existence of such epigenetically driven, drug-tolerant cell states with enriched EMT signatures [71,72]. In one particular study, a mesenchymal drug-tolerant state has been observed to function as an important intermediate stage, enabling initially-*EGFR*^T790M^-negative non-small-cell lung cancer cells to acquire this “gatekeeper” mutation following treatment with EGFR inhibitors [71].

Recent studies also suggest that the EMT program contributes to the establishment of an immunosuppressive tumor microenvironment and thereby confers resistance to immunotherapies (Fig. 3C) [73]. EMT-mediated resistance to immunotherapies seems to be acquired through both cell-autonomous and non-autonomous mechanisms. From the perspective of cancer cells, induction of an EMT program in carcinoma cells has been shown to reduce susceptibility to cytotoxic T cell-mediated lysis, possibly due to the reduced vulnerability to apoptosis in quasi-mesenchymal cell states [74,75]. Consistently, naturally arising mesenchymal cells from MMTV-PyMT mouse mammary tumor model have been found to express markedly lower levels of MHC-I molecules and β2-microglobulin compared with their epithelial counterparts, yielding an immunoevasive phenotypic state [76]. In lung and breast carcinoma cells, upregulation of the ZEB1 EMT-TF has been shown to induce the expression of PDL1, an immune-inhibitory checkpoint ligand that suppresses the function of activated T cells through binding to its cognate PD-1 receptor expressed by the latter [77,78].

In addition, induction of EMT has been shown to remodel the tumor microenvironment, helping to convert it into an immunosuppressive state (Fig. 3C). Thus, Snail-induced EMT in melanoma cells has been shown to increase the infiltration of immunosuppressive regulatory T cells in the tumor microenvironment, partly through an increased secretion of TGF-β and thrombospondin-1 by the quasi-mesenchymal cancer cells [79]. Similar observations have been reported in breast cancer models in which tumors initiated from more epithelial MMTV-PyMT carcinoma cells contained more M1 (anti-tumor) macrophages and CD8^+^ T cells. In contrast, tumors arising from more mesenchymal cells contained more regulatory T cells and M2 (pro-tumorigenic) macrophages. At the same time, these tumors contained fewer CD8^+^ T cells, most of which showed markers of functional exhaustion. Importantly, in this breast cancer model, a minority of quasi-mesenchymal cancer cells within a tumor were able to induce the immunosuppressive microenvironment and protect the more epithelial cancer cells residing in the same tumor from immune attack [76]. These studies highlight the critical need to acquire further insights into the influences of EMT programs on modulating tumor microenvironment.

In summary, these diverse observations help to explain how EMT-induced CSCs can serve as a cell population that limits the efficacy of currently employed anticancer therapies. It is highly plausible that many therapeutic regimens largely target the bulk populations of more epithelial non-CSCs, while being unable to eliminate the minority subpopulations of CSCs. In the context of immunotherapy, an EMT-induced immunosuppressive tumor microenvironment may protect not only the mesenchymal CSCs but also epithelial non-CSCs. Moreover, the surviving CSCs, given their tumor-initiating capacity, may be able to initiate new tumors, eventually leading to clinical relapse.

### Complexity of the epithelial-mesenchymal phenotypic spectrum

During the multistep tumor progression of carcinomas, EMT programs operate in cells residing both at the very beginning stages of this process (involving normal epithelial cells), and the final stage (involving fully malignant carcinoma cells). These observations suggest that a core EMT program also participates in all the cell populations existing in the intermediate stages of multi-step tumor progression, i.e., cell populations lying between these two endpoints. This core EMT program might collaborate with other cellular programs to generate a spectrum of distinct cell-biological states along the E versus M axis. These intermediate cell states, displaying distinct cellular behaviors, such as differing powers of invasiveness and drug resistance, may facilitate cancer cell proliferation and dissemination occurring at different stages of tumor progression [64]. These complex behaviors suggest a variety of alternative versions of the EMT program, and conversely complicate attempts to rationalize cancer cell behavior during multi-stage progression in terms of a single, uniformly expressed EMT program.

Indeed, several dimensions of complexity contribute to the phenotypic heterogeneity generated by the EMT program. First, although the EMT program is executed by a relatively small number of master regulators, e.g., the SNAIL, TWIST, SLUG and ZEB1 EMT-TFs; these proteins, acting in various combinations, are not equally potent in repressing epithelial properties and inducing mesenchymal features [80,81]. In addition, they display many non-redundant functions. For example, as mentioned above, while SLUG plays an essential role in maintaining stemness in normal gland-reconstituting mammary stem cells, SNAIL is the EMT-TF that is utilized in breast cancer cells to generate CSCs and trigger metastasis [29]. Second, different developmental origins may dictate distinct responses to expressed EMT-TFs, yielding entrance into diverse phenotypic states arrayed along the E versus M spectrum. Accordingly, the same EMT-TF may elicit distinct cellular responses in different carcinoma types. In support of this notion, despite the observation that SNAIL is critical for promoting metastasis in the PyMT mouse model of breast cancer, this particular EMT-TF seems to be dispensable for metastasis in the KPC mouse pancreatic cancer model [29,82,83]. Interestingly, ZEB1 has been shown to operate as a key factor driving metastasis in the latter model [36]. Third, activation of EMT programs is induced by converging heterotypic signals *in vivo*. Given the heterogeneous cellular microenvironment within a tumor, individual cancer cells in various locations may reside at different distances from signal-emitting stromal cells, encounter different levels of EMT-inducing cytokines, and experience different degrees of hypoxia [84]. These different combinations of contextual signals might induce cancer cells to enter distinct EMT intermediate states along the E-M axis. Among other implications, this suggests topological localization of EMT-induced traits within individual tumors. For example, by analyzing head-and-neck squamous cell carcinomas at the single-cell level, cells with partial EMT features have been found to spatially localize to the leading edge of primary tumors and facilitate invasion [43].

At the mechanistic level, we still lack coherent understanding of the molecular and biochemical mechanisms inducing cells to enter various states arrayed along the epithelial-mesenchymal phenotypic spectrum. We do know that a complex regulatory network that orchestrates EMTs and modulates the expression and function of EMTTFs at multiple mechanistic levels, including transcription, post-transcription, epigenetic modification, alternative splicing, protein stability and subcellular localization [1,81]. For example, the EMT-TFs ZEB1 and SNAIL form double-negative feedback loops with miR-200 and miR-34, respectively, which is thought to maintain epithelial homeostasis under physiological conditions [85–88]. Several transcription factors, including ELF5, GRHL2, OVOL1/2 and p53, have also been found to function as “guardians of the epithelial phenotype” in certain contexts by suppressing the expression of specific EMT-TFs [89–92]. In addition, cells residing in epithelial and mesenchymal states display distinct RNA splicing programs [93]. Some of the splicing factors, such as ESRP family members, are associated with the epithelial phenotype and regulate production of key transcripts whose encoded products are involved cell-cell adhesion, cell-matrix adhesion, and invasion [94]. Other splicing factors, like QKI and RBFOX2, are found to be upregulated in the more mesenchymal cell state and suppress epithelial properties [95,96]. Several chromatin modifiers, including HDAC1/2, LSD1 and components of PRC2 complex, are recruited by certain EMT-TFs to their target promoters. These epigenetic regulators are plausibly involved in forming epigenetic regulatory loops, that may control the interconversions between different intermediate EMT states [97–99]. In addition, at the protein level, ubiquitin-mediated degradation and phosphorylation-induced subcellular localization of EMT-TFs have been shown to regulate the EMT process. The ubiquitin ligases and phosphatases participating in this process can be induced by a variety of intracellular signaling channels, such as those involving the WNT, MAPK and DNA damage pathways [100,101]. In addition, the establishment and maintenance of mesenchymal/CSC states require specific contextual signals and distinct intermediate EMT states are plausibly maintained in different niches within the tumor microenvironment. For example, TGF-β has been shown to induce EMT and maintain the mesenchymal cell state in many cell systems and the mechanisms explaining how TGF-β activates the EMT program have been reviewed elsewhere [102]. However, induction of EMT by TGF-β appears to need an appropriate intracellular context and thus is not universal for all the cell types or cell states [103]. A mesenchymal/CSC state can also be maintained by a juxtacrine signaling from monocytes and macrophages, or by prostaglandin E_2_ (PGE_2_) secreted from mesenchymal stem cells [104,105]. It remains to be determined whether these mechanisms are also functional *in vivo*, and whether the mesenchymal/CSC state generated through these mechanisms represent discrete intermediate states arrayed along the epithelial-mesenchymal axis.

All this explains the ongoing need to construct a systematic framework of EMT regulation in order to incorporate these diverse mechanisms into a single over-arching scheme. Such a scheme will lay the foundation for answering four major categories of questions: (1) In a particular cancer type, how many intermediate phenotypic states exist between the fully epithelial and the fully mesenchymal states? Are these different phenotypic EMT states shared among different cancer types? (2) What genes constitute the core gene circuit in each of these intermediate states? Can residence in these metastable phenotypic states be maintained over multiple cell generations? Do the E-to-M conversion requires a cell division? (3) For each of these EMT states, which specific extracellular signals are sufficient to induce metastable residence of carcinoma cells in these multiple alternative phenotypic states? (4) What are the precise functional contributions of these intermediate states to the multistep cancer progression?

### Concluding remarks and future directions

Major conceptual advances about the EMT program in cancer progression have provided us with new insights into the biological basis of tumor malignancy, including (1) the role of EMT programs in promoting cancer cell dissemination in both “single cell migration” and “collective migration” models, (2) the connection between EMT program and the CSC state, and (3) the contribution of EMT programs to the acquired resistance to chemo- and immuno-therapies. Recent findings have highlighted the dynamic and plastic nature of the EMT program, suggesting the existence of diverse phenotypic cell states arrayed along the E-to-M spectrum. Nonetheless, we still lack a systematic framework to identify all the states orchestrated by EMT programs and the responsible intracellular control circuits. Future research will be needed to explore these cell-biological programs at a higher resolution, ideally at the single-cell level, doing so in order to generate a complete map of all the intermediate cell states between the two end points of the E to M axis. In addition, to fully understand the functional contribution of EMT programs in cancer metastasis and relapse, it will be necessary to develop more sophisticated mouse models that enable real-time monitoring of the residence of cancer cells in various EMT-induced states and/or lineage-tracing of the cancer cells that have entered certain EMT states during the course of tumor development.

Given the pleiotropic roles of the EMT program in the invasion-metastasis cascade and the acquisition of therapeutic resistance, the development of novel therapies targeting this cellular program is clearly desirable. Theoretically, at least three strategies for targeting this program seem worthy of further exploration:

(1) Specifically targeting cancer cells that have undergone EMT and display mesenchymal/CSC features. Along this line, the receptor tyrosine kinase AXL has been found to associate with EMT induction and confers resistance to EGFR-targeted therapy in a cohort of NSCLC patients [70]. When treated with both EGFR and AXL inhibitors, NSCLC cells could no longer develop the EMT-induced resistance to treatment by erlotinib (an EGFR inhibitor) in a mouse xenograft model [70]. The first AXL-specific inhibitor, BGB324, has entered clinical trials recently [106,107]. In addition, two recent studies demonstrated that the mesenchymal cell state depends on a druggable lipid-peroxidase pathway in a variety of cancer types. Inhibition of GPX4, a selenocysteine-containing enzyme that plays a central role in this pathway, induces ferroptotic cell death specifically in mesenchymal cell populations [108,109].
(2) Reverse the process of EMT at certain stages of tumor development by differentiation-inducing therapies. For example, cholera toxin and forskolin have been found to enhance protein kinase A signaling, triggering the process of MET and thus reducing the invasiveness and tumor-initiating abilities of mammary tumor cells [110]. The clinical implementation of this strategy needs to be designed with great care, in light of the fact that the MET process may actually promote colonization, the last step of the invasion-metastasis cascade.
(3) Inhibiting the plasticity of cancer cells and preventing the EMT induction. EMT can be prevented by targeting the signaling processes that induce and subsequently maintain certain mesenchymal states. From this perspective, TGF-β inhibitors are the most intensively investigated anti-EMT compounds. One particular TGF-β inhibitor, termed LY2157299, has entered clinical trial recently [111]. However, it should be noted that TGF-β has multifaceted effects on cancer cells in a context-dependent manner. Thus it remains to be determined which particular clinical indications suggest implementation of this treatment. Nonetheless, while the current therapeutic strategies targeting the EMT program are still rudimentary, this overall direction represents an attractive avenue for the future development of truly effective therapies designed to manage high-grade tumor malignancies.
